# The “Christian Hospital”

**Published:** 1903-08

**Authors:** 


### THE "CHRISTIAN HOSPITAL."

ALONG with many others we have received a pressing and cordial invitation to become a member of the staff of physicians, surgeons and specialists of the Christian Hospital of Chicago. Proceeding further, however, it was ascertained that this invitation was not a testimonial to our strenuous life of self-sacrificing devotion to the cause of uplifting man from his fallen estate, but was procurable only on the purchase of a certificate at a sum ranking from $5 to $25, depending upon the degree of artistic embellishment of the document. With the high-priced certificates, a gold lapel button was prescribed bearing the device of the Geneva cross, and the legend Christian Hospital, Chicago. By this means those who were not so fortunate as to see the certificate in the doctor's office, could at least perceive that the holder was a man of parts as he mingled with the public.

One of the chiefest recommendations of this certificate is its enabling power to impress the waiting patient. It adds a touch of rococo to the dinginess of the village doctor's sanctum, offsetting the dull tones of the crippled chairs, rusty stove barnacled with dead quids, and that base but useful object of salivary ejaculation the spit-box. But even as examples of florid ornamentation can they compete with brewery calendars and the alluring charms of Koch's Dandruff Cure young ladies, which (not the young ladies) are free for the asking?

Half-soled, freshly greased and displayed for rustic sale, we recognize in the Christian Hospital the exploded Niles, Michigan, fake which we exploited some years ago. A difference is noted in the fresh accretion of titles and degrees attached to the names of the exploiters. Perhaps if the Joliet Penitentiary or the Dwight establishments were empowered to give the baccalaureate degree, this might be added with more truth than flattery.

The medical profession must all be members of the Mark family. The impositions that are worked off on them seem to establish the relationship. But there are some schemes too palpable and gauzy to deceive even the eclectics—and the Christian Hospital is one of the most diaphanous.

---

DR. ORPHEUS EVERTS, of the Cincinnati Sanitarium, is dead at an advanced age. He was well known as an alienist and a writer on this subject.

---

DR. JOHN E. WALKER, a well-known physician of Columbus, died there July 12. He had been in bad health for some time.

